# Correction of Abeditehrani, H., Dijk, C., Sahragard Toghchi, M., & Arntz, A. (2020). Integrating Cognitive Behavioral Group Therapy and Psychodrama for Social Anxiety Disorder: An Intervention Description and an Uncontrolled Pilot Trial

**DOI:** 10.32872/cpe.7727

**Published:** 2021-12-23

Correction note to:

AbeditehraniH.DijkC.Sahragard ToghchiM.ArntzA.
(2021). Integrating cognitive behavioral group therapy and psychodrama for social anxiety disorder: An intervention description and an uncontrolled pilot trial.
Clinical Psychology in Europe, 2(1), e2693. 10.32872/cpe.v2i1.2693

In the originally published version of the above mentioned article, there was a typographical error in Table 4. In the second row ("LSAS"), an incorrect value in the "*p*" column was provided (.19). Instead, the correct data for "LSAS" under the "*p*" column is: .019. This change has no effect on the conclusions drawn in the article as the authors had already explained correctly that there was a significant decrease (*p* = .019) in social anxiety symptoms assessed with the LSAS. The corrected table can be found below (see Table 4). The authors apologize for any inconveniences caused.

**Table 4:** Pretest and Posttest Comparison for the CBPT Intervention

Scale	Pre		Post		*t* (4)	*M* difference [CI 99%]			Cohen’s *d*	Hedges*’ g*
	*M*	*SD*	*M*	*SD*		*LL*	*UL*	*p*		
BFNE	35.60	7.02	28.40	4.10	2.86	-4.39	18.79	.046	1.03	0.82
LSAS	99.40	16.99	58.40	24.81	3.82	-8.44	90.44	.019	2.41	1.93
SADS	14.40	5.64	11.80	7.73	1.31	-6.56	11.76	.261	0.46	0.37
PAS	133.20	13.88	131.60	17.21	0.20	-34.67	37.87	.849	-0.12	-0.09
OPQ	56.20	23.18	35.80	16.63	3.22	-8.74	49.54	.032	0.88	0.70
OCQ	64.80	23.47	47.00	26.67	5.95	4.03	31.57	.004	0.76	0.61
BDI	19.60	5.86	12.60	8.20	2.03	-8.82	22.82	.111	1.19	0.96
QOLI	29.40	21.31	36.00	25.17	-0.88	-41.13	27.93	.429	0.31	0.25

*Note*. Observed Means *(M)* and Standard Deviations *(SD)* for the Pre and Post assessment points; results of *t*-test analyses *(t, p-value)* and effect sizes Cohen’s *d* and Hedges’ *g*. BFNE = Brief Fear of Negative Evaluation; LSAS = Liebowitz Social Anxiety Scale; SADS = Social Avoidance and Distress Scale; PAS = Personal Attitude Scale-II; OPQ = Social cost and probability by the Outcome Probability Questionnaire; OCQ = Outcome Cost Questionnaire; BDI = Beck Depression Inventory; QOLI = Quality of Life Inventory. Cohen’s *d* was estimated as *d* = (mean pre-post change)/(pretest *SD*). Hedges’ *g* was calculated as follows: *g* = J*d, with *d* = Cohen’s *d*; J = (1 – 3/(4*df-1)); *df* = *N*-1. The sign of the effect size was chosen so that a positive effect size indicates improvement and negative effect size represents worsening.

